# A General Catalytic Enantioselective Transfer Hydrogenation Reaction of β,β-Disubstituted Nitroalkenes Promoted by a Simple Organocatalyst

**DOI:** 10.3390/molecules21081000

**Published:** 2016-07-30

**Authors:** Luca Bernardi, Mariafrancesca Fochi

**Affiliations:** Department of Industrial Chemistry “Toso Montanari”and INSTM RU Bologna, Alma Mater Studiorum, University of Bologna, V. Risorgimento 4, 40136 Bologna, Italy; luca.bernardi2@unibo.it (L.B.); mariafrancesca.fochi@unibo.it (M.F.); Tel.: +39-051-209-3653 (L.B.); +39-051-209-3626 (M.F.)

**Keywords:** asymmetric catalysis, Hantzsch ester, H-bond, nitroalkanes, nitroalkenes, organocatalysis, reduction, thiourea, transfer hydrogenation

## Abstract

Given its synthetic relevance, the catalytic enantioselective reduction of β,β-disubstituted nitroalkenes has received a great deal of attention. Several bio-, metal-, and organo-catalytic methods have been developed, which however are usually applicable to single classes of nitroalkene substrates. In this paper, we present an account of our previous work on this transformation, which implemented with new disclosures and mechanistic insights results in a very general protocol for nitroalkene reductions. The proposed methodology is characterized by (i) a remarkably broad scope encompassing various nitroalkene classes; (ii) Hantzsch esters as convenient (on a preparative scale) hydrogen surrogates; (iii) a simple and commercially available thiourea as catalyst; (iv) user-friendly procedures. Overall, the proposed protocol gives a practical dimension to the catalytic enantioselective reduction of β,β-disubstituted nitroalkenes, offering a useful and general platform for the preparation of nitroalkanes bearing a stereogenic center at the β-position in a highly enantioenriched form. A transition state model derived from control kinetic experiments combined with literature data is proposed and discussed. This model accounts and justifies the observed experimental results.

## 1. Introduction

**Scheme 1:**
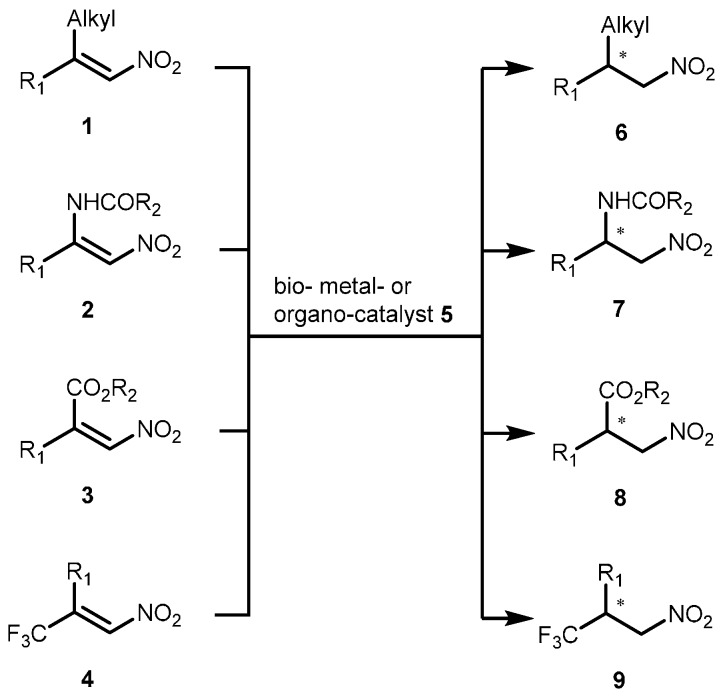
Nitroalkene substrates **1**–**4** employed in catalytic enantioselective reductions, and corresponding nitroalkane products **6**–**9**.

The enantioselective reduction of pro-chiral β,β-disubstituted nitroalkenes is a powerful synthetic transformation. It provides a straightforward access to optically active nitroalkanes carrying a configurationally stable stereogenic center at the β-position of the nitro group. These compounds can be easily converted to broadly useful chiral building blocks (e.g., enantioenriched β-chiral amines) exploiting the renowned synthetic versatility of the nitro moiety [1,2,3]. Accordingly, this reaction has received considerable attention from the synthetic chemistry community, leading to several efficient protocols based on different catalytic approaches and encompassing various nitroalkene substrates **1**–**4**, providing access to enantioenriched nitroalkanes **6**–**9** (Scheme 1).

“Ene”-reductases, mainly of the OYE (Old Yellow Enzyme) family, have been applied to the biocatalytic enantioselective reduction of β-alkyl-β-aryl nitroalkenes **1** [4,5,6,7,8]. In fact, α-methyl-β-nitrostyrene is a benchmark substrate for “ene”-reductases and stereocomplementary enzymes, guaranteeing access to both enantiomers of the reduced product, are known [8]. However, variations in the aryl and alkyl moieties of substrates **1** have only been partially addressed. Concurrently, enantioselective metal-catalyzed reductions of β,β-disubstituted nitroalkenes have been developed, employing different chiral complexes based on Cu, Rh, and Ir in hydrosilylation, transfer hydrogenation, and hydrogenation reactions [9,10,11,12,13,14,15,16,17,18,19,20]. Protocols featuring very broad substrate scopes encompassing not only a variety of β-alkyl-β-aryl and β,β-dialkyl nitroalkenes **1** [9,10,11,12,13,14,15,16], but also their β-amido [17,18,19] and β-carbonyl [20] counterparts **2**–**3** are available. A Lewis base catalyzed hydrosilylation reaction with substrates **2** has also been reported [21].

**Scheme 2:**
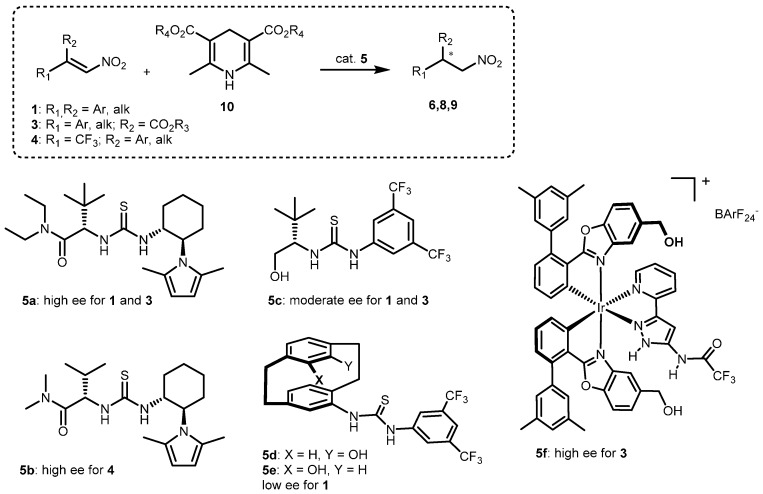
Organocatalysts **5a**–**f** employed in transfer hydrogenation reactions of β,β-disubstituted nitroalkenes with Hantzsch esters, and their substrate scope.

An alternative strategy to enantioselective reduction that has gained great attention, due to its elegance, simplicity, and practical features (at least on a preparative scale), is the organocatalytic transfer hydrogenation reaction with Hantzsch esters **10** as hydrogen surrogates [22,23,24,25,26,27,28]. In this context, exploring the notion of the efficient coordination of the nitro group by double hydrogen bond donors [29,30,31], thioureas, and related catalysts **5a**–**f** (Scheme 2) have been found to be efficient in the promotion of the enantioselective reduction of β,β-disubstituted nitroalkenes **1**, **3**, **4** with Hantzsch esters **10** [32,33,34,35,36,37,38,39,40]. Catalyst **5a**, a thiourea resulting from the combination of an *N*,*N*-diethyl *tert*-butylglycinamide residue on one side and an elaborated *trans*-1,2-cyclohexanediamine on the other, has been successfully employed in the reaction with nitroalkenes **1** and **3** [32,33]. The related thiourea **5b**, keeping the same structural features of **5a** but bearing a different amino-acidic component (*N*,*N*-dimethylvalinamide), proved instead to be efficient in the reduction of β-trifluoromethylnitroalkenes **4** [34]. The simpler structure **5c**, wherein the cyclohexanediamine part was replaced by the 3,5-bis(trifluoromethyl)phenyl group, and carrying an alcohol in place of the amide moiety, was applied to substrates **1** and **3** giving results worse than **5a** in terms of enantioselectivities [35]. The unrelated paracyclophane catalysts **5d** and **5e** were also tested in the reaction with a nitroalkene **1**, but gave only low enantioselectivity [36]. Conversely, a chiral-at-metal pyrazole-amide double hydrogen bond donor **5f** was applied very successfully to the transfer hydrogenation reaction with nitroalkenes **3** [37].

**Scheme 3:**
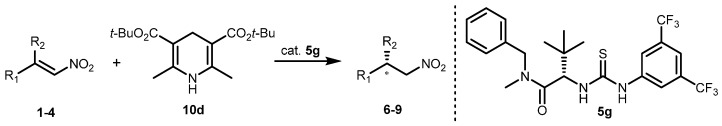
A general enantioselective transfer hydrogenation reaction of β,β-disubstituted nitroalkenes **1**–**4** with Hantzsch ester **10d** catalyzed by the simple thiourea **5g**.

Herein, we report a general enantioselective transfer hydrogenation reaction of β,β-disubstituted nitroalkenes with *tert*-butyl Hantzsch ester **10d**, catalyzed by the simple and commercially available Jacobsen type thiourea catalyst **5g** [41,42,43] and applicable to all nitroalkene classes **1**–**4** (Scheme 3). Catalyst **5g**, originally developed in the frame of the Strecker reaction, is considerably simpler to synthesize than **5a,b** or **5f**, the previous most successful structures, that moreover were only applied to some nitroalkene classes (Scheme 2). In this paper, we first detail our previous development of the reaction with β-trifluoromethyl nitroalkenes **4** [39]. Then, providing experimental evidence on the mode of action of catalyst **5g** in this transformation, we demonstrate its remarkable tolerance to variations in the substituents of the nitroalkene substrate (i.e., R_1_ and R_2_ in Scheme 3). By slightly varying reaction conditions, we show its application to nitroalkenes **2** [40], **1** and **3**, ultimately providing a very general and broadly useful protocol for enantioselective transfer hydrogenation reactions of β,β-disubstituted nitroalkenes based on a readily available catalyst.

## 2. Results and Discussion

### 2.1. Optimization and Development of the Transfer Hydrogenation Reaction with β-Trifluoromethylnitroalkenes ***4***

**Scheme 4:**
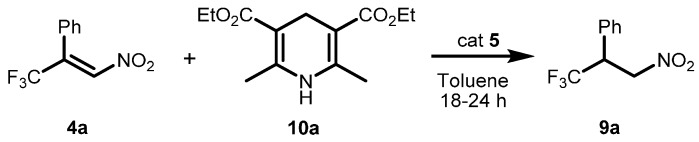
Test transfer hydrogenation reaction used to identify an efficient catalyst **5**.

We started our investigation by studying the H-bond driven transfer hydrogenation reaction of β-trifluoromethyl nitroalkene **4a** with Hantzsch ester **10a** (Scheme 4) testing various typical H-bond donor catalysts (thioureas, ureas, squaramides, diols, phosphoric acids, etc.).

Even if most catalysts **5** were able to afford the desired β-trifluoromethyl nitroalkane **9a** with good conversions (Table 1, entries 1–7) only the 1,2-diaminocyclohexane derived amido-thiourea **5o** gave a promising enantioselectivity in the reaction (entry 8). Accordingly, variations in the two thiourea *N*-substituents were undertaken exploiting the modularity of this catalyst structure.

Catalyst **5p** (entry 9) bearing the same 1,2-diaminocyclohexane moiety as **5o** afforded the product with lower conversion and very poor and opposite selectivity, showing that enantioselectivity is driven by the amide portion of the catalyst. Indeed, the simpler amido-thiourea catalyst **5g** (entry 10) afforded results even better than **5o**.

Whereas the utility of a 1,2-cyclohexanediamine derived catalysts (i.e., **5a**, Scheme 2) for the asymmetric reactions of nitroalkenes with Hantzsch esters had been previously reported [32,33], the notable outcome of the simpler structure **5g** was an unanticipated yet gratifying result. Having identified catalyst **5g** as the optimal, a screening of reaction conditions and Hantzsch esters **10** was undertaken, performing the reactions under more concentrated conditions and at 40 °C, to ensure full conversion (Table 2). The methyl, *iso*-butyl, and *tert*-butyl Hantzsch esters **10b**–**d** were tested and compared with **10a** (entries 1–4). The *tert*-butyl derivative **10d** outclassed the other dihydropyridines **10a**–**c** in term of enantioinduction, affording **9a** in 89% ee and for this reason it was selected for further exploration. The superior behavior the *tert*-butyl Hantzsch ester **10d** compared to other esters, a common feature to many organocatalytic reactions, had been previously rationalized invoking not only increased nonbonding interactions between the Hantzsch ester ring and the catalyst-substrate complex, but also electronic factors related to the boat-like conformation of the *tert*-butyl Hantzsch ester **10d** [23]. However, it is also important to underline that *tert*-butyl ester **10d** features improved solubility in apolar solvents, compared to **10a**–**c**. Next, toluene was confirmed as the most appropriate solvent after a short screening (entries 4–7), and a satisfactory 94% ee value was accomplished by cooling the reaction mixture to −20 °C (entry 8).

A variation of the amide *N*-substituents in catalyst **5** was then explored, by using the closely related thioureas **5q** and **5r** in the transfer hydrogenation reaction, at −20 °C in toluene. The *N*,*N*-diethyl derivative **5q** performed rather poorly, while the *N*-methyl-*N*-benzhydryl amide **5r** did not give any improvement compared to **5g** (entries 9–10). The use of α,α,α-trifluorotoluene as solvent provided slightly better results than toluene, when catalyst **5g** was employed (entry 11 vs. entry 8). Unluckily, a lower catalyst loading gave a small decrease in the enantiomeric excess of the product **9a** (entry 12). Reverting to 10 mol % loading, slightly better results were achieved by lowering reaction concentration (entry 13).

**Scheme 5:**
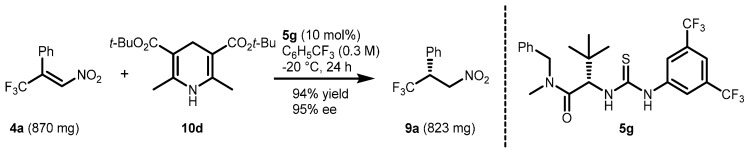
Optimized reaction on preparative scale.

These conditions, namely catalyst **5g** (10 mol%) Hantzsch ester **10d**, α,α,α-trifluorotoluene as solvent, and −20 °C as reaction temperature, were applied on a preparative scale (Scheme 5) obtaining product **9a** in 94% yield, with a small erosion in the enantioselectivity compared to the optimization scale reaction (95% instead of 97%).

**Scheme 6:**
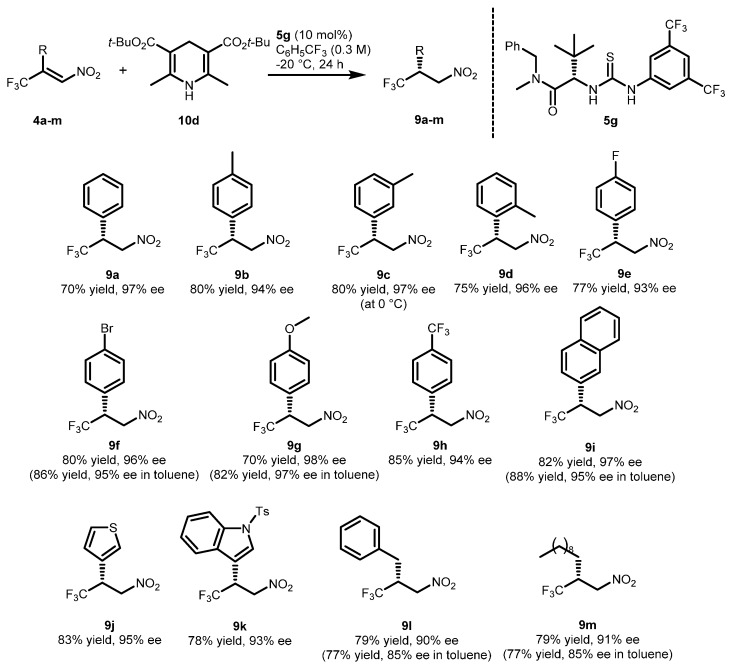
Scope of the transfer hydrogenation reaction of β-trifluoromethyl nitroalkenes **4**.

A study of the scope of the reaction was undertaken (Scheme 6). Similarly to derivative **4a**, the reactions with different substrates **4b**–**h** bearing aromatic rings substituted at different positions with either electron donating or electron withdrawing groups furnished a series of β-aryl-β-trifluoro nitroalkanes **9b**–**h** with excellent results (>70% yield and >93% ee). Very good yields and enantioselectivities (93%–97% ee) can also be achieved when a 2-naphthyl and two heteroaromatic substituents are collocated in substrates **4i**–**k**. Notably, the optimized reaction conditions could also be applied successfully to substrates **4l** and **4m** bearing aliphatic chains, which furnished the expected adducts **9l** and **9m** with very good results. The benefit of using α,α,α-trifluorotoluene as reaction medium with respect to toluene was demonstrated by performing few reactions using toluene as the solvent (Scheme 6, results in brackets): in all the examined cases, the results in terms of enantioselectivity were slightly lower.

### 2.2. Extension of the Transfer Hydrogenation Reaction Catalyzed by ***5g*** to Other Nitroalkenes ***1***–***3***

**Figure 1:**
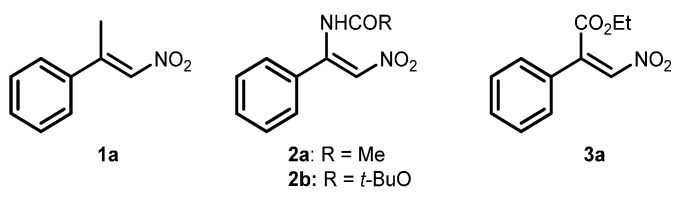
Additional nitroalkenes **1**–**3** studied.

The relevant results obtained in the transfer hydrogenation reaction of β-trifluoromethyl nitroalkenes **4** catalyzed by thiourea **5g** prompted us to verify the applicability of the methodology to other readily accessible β,β-disubstitued nitroolefins such us (*E*)-1-nitro-2-phenyl-1-propene **1a**, β-amino nitroolefines **2a** and **2b**, and β-nitroacrylate **3a** (Figure 1).

Substrates **1a** and **3a** had been previously studied and organocatalytic protocols based on thioureas are present in the literature (see Scheme 2); on the contrary, prior to our work [40], β-amino nitroolefines **2** were utilized only in asymmetric hydrosilylation [21] or in metal-catalyzed asymmetric hydrogenations [17,18,19].

**Scheme 7:**
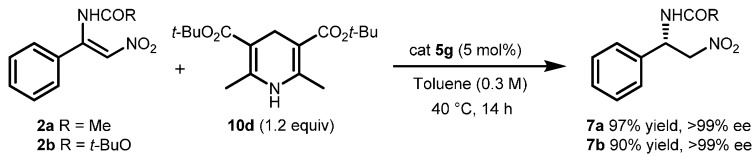
Catalytic enantioselective transfer hydrogenation reactions with substrates **2a** and **2b**.

To our delight, after carefully adjusting the reaction conditions, thiourea **5g** was able to promote the transfer hydrogenation reaction of **2a** and **2b** in the presence of Hantzsch ester **10d** as hydrogen donor, affording the corresponding β-amino nitroalkanes **7a** and **7b** in 97% and 90% yield respectively, with complete enantioselectivity (ee >99%) regardless of the protecting group installed on the amine function (Scheme 7). Although a higher reaction temperature was required with these substrates **2** compared to the trifluoromethyl derivatives **4**, presumably due to stereoelectronic effects rendering amido substrates **2** less reactive than **4**, full enantioselectivity was observed even at a lower (5 mol %) catalyst loading.

**Scheme 8:**
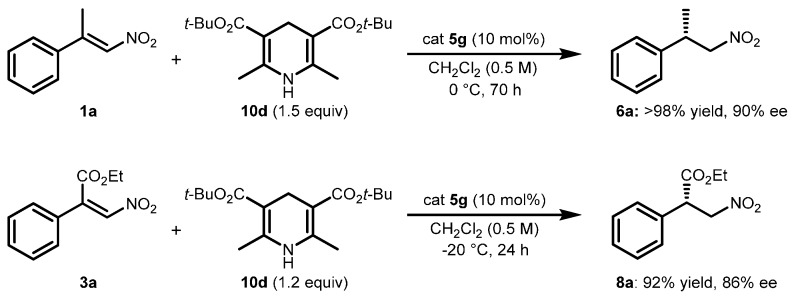
Catalytic enantioselective transfer hydrogenation reactions with substrates **1a** and **3a**.

Eager to further verify the capacity of catalyst **5g** in inducing enantioselectivity in the reaction of β,β-disubstitued nitroolefin substrates with Hantzsch ester **10d**, we moved to investigate substrates **1a** and **3a**. A short screening of reaction conditions (solvent, dilution, temperature, amount of Hantzsch ester **10d**), indicated dichloromethane as a more suitable solvent for these substrates, and 0 and −20 °C as optimal reaction temperatures for **1a** and **3a**, respectively. Under these newly found conditions, catalyst **5g** was indeed able to provide the corresponding nitroalkanes **6a** and **8a** in high yields and enantioselectivities, as depicted in Scheme 8.

### 2.3. Proposed Reaction Model for the Transfer Hydrogenation Reactions Catalyzed by ***5g*** with Nitroalkenes

**Scheme 9:**
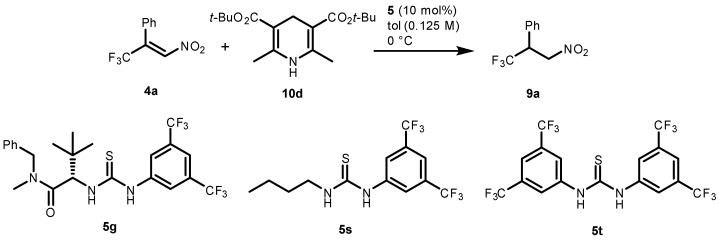
Control experiments using different thiourea catalysts.

Keeping the activation of nitro compounds by thioureas as the preliminary reasonable assumption, we aimed at gaining some insights on the mode of action of catalyst **5g** in the reactions. We first performed some simple control experiments, comparing in the reaction between **4a** and **10d** the activity of catalyst **5g** vs. simpler achiral thiourea derivatives **5s** and **5t** (Scheme 9).

**Figure 2:**
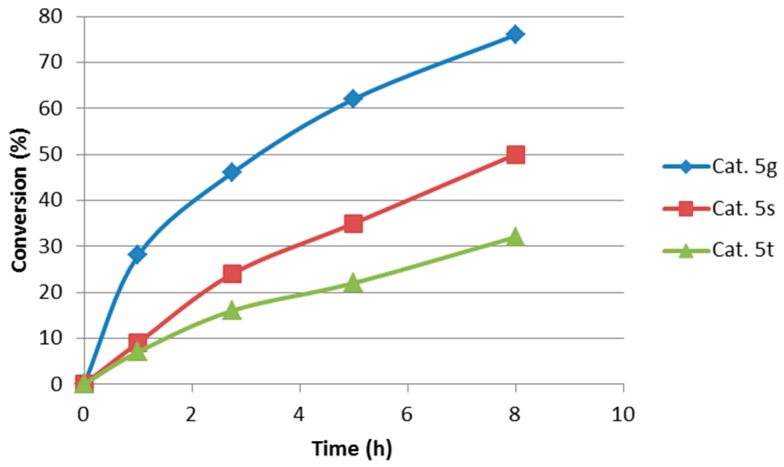
Evolution over time (^19^F-NMR) of the reactions performed with catalysts **5g**, **5s**, and **5t**.

The evolution of the reactions was conveniently monitored by ^19^F-NMR spectroscopy. Whereas thiourea **5s** should feature a similar acidity to **5g** (p*K*_a_ ca. 12.57 in DMSO [44]), **5t** bearing two aryl substituents is considerably more acidic (p*K*_a_ ca. 8.5 in DMSO [44]). Considering only the coordination of the nitro group by the thiourea moiety acting as a general acid catalyst, and neglecting conformational effects, an increase in acidity should roughly correspond to an increase in catalyst activity [45]. However, as shown in Figure 2, catalyst **5g** is considerably more efficient in reaction promotion than **5s** and **5t**. Furthermore, the less acidic **5s** was found to be slightly more effective than **5t**.

These reactions were found to obey pseudo-second order kinetics according to the following equation [46]:

(1) $$\ln(\frac{{\left[4a\right]}_{0}\;\times \;\left({\left[10d\right]}_{0}-\left[9a\right]\right)}{{\left[10d\right]}_{0}\times \left({\left[4a\right]}_{0}-\left[9a\right]\right)})=\left({\left[10d\right]}_{0}-{\left[4a\right]}_{0}\right)\times k_{\text{obs}}t$$

where:

[**4a**]_0_ = 0.125 M; [**10d**]_0_ = 0.1875 M; [**9a**] = (0.125 × conversion) M (2)

**Figure 3:**
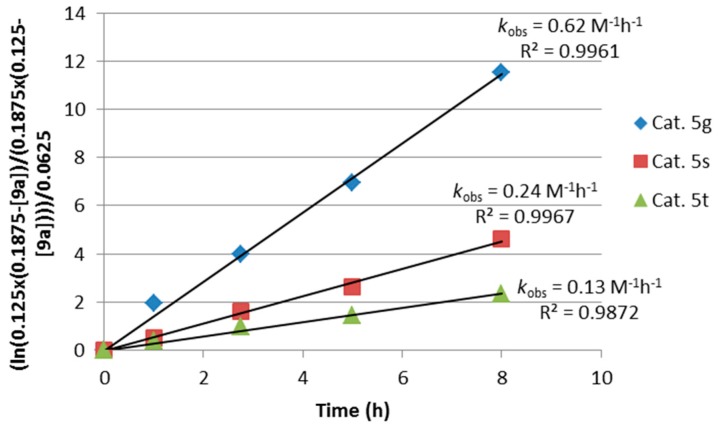
Pseudo-second order rate constants for the three catalysts **5g**, **5s**, and **5t**.

The straight lines reported in Figure 3 display good correlation coefficients. Their slopes are the rate constant *k*_obs_ (in M^−1^·h^−1^) for the reactions, from which a more quantitative comparison of the activity displayed by the three catalysts **5g**, **5s**, and **5t** can be deduced.

**Scheme 10:**
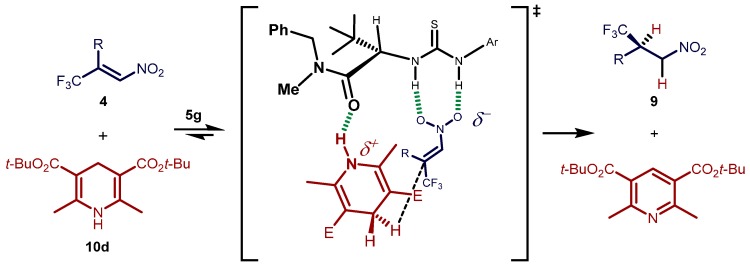
Transition state model for the reduction of nitroalkenes **4** catalyzed by **5g**.

Together with the previously determined mode of action of catalyst **5g** in the Strecker reaction [41,42], these experiments suggest that the amide moiety of this catalyst effectively participates in the coordination/stabilization of a reaction transition state (TS) leading to the major enantiomer of product **9**. The pseudo-second order kinetics followed by the reactions (Figure 3) indicates as a first approximation that interactions prior to TS are not relevant (i.e., Curtin-Hammett control). Thus, it can be surmised that, along with the coordination of the Lewis acidic thiourea moiety to the negatively charged nitro moiety, the amide oxygen acts as a Lewis base in this TS, coordinating the N-H proton of the Hantzsch ester having a positive charge density. Applying the previously determined most stable conformation of catalyst **5g** [41], these considerations result in the model depicted in Scheme 10. Overall, a dipolar TS is effectively stabilized by the electrostatically complementary functionalities present in the catalyst structure, in a way reminding the mode of action of some macromolecular enzymes [47]. A coordination of both dipole charges (negative at the nitro moiety and positive at the Hantzsch ester) might be more efficient than an exclusive coordination of the negative part of the dipole (nitro moiety), justifying the lower activity offered by “mono-functional” catalysts **5s** and **5t**. After TS, an irreversible proton-transfer follows, leading to the products **9** and the pyridine co-product. We have previously demonstrated [40], using an α-substituted nitroalkene giving a pro-chiral nitronate intermediate, that catalyst **5g** is not able to exert significant stereocontrol in this proton-transfer process [48,49].

**Scheme 11:**
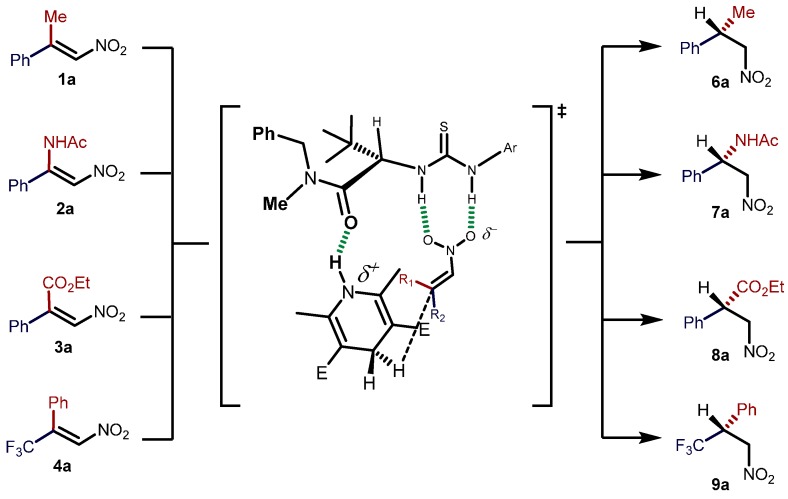
Attack of the hydride to the same pro-chiral face of **1**–**4** according to the TS model.

The soundness of this model is confirmed by computational work on the transfer hydrogenation reaction with the related catalyst **5b** [34]. In line with the established mode of action of thiourea catalysts in this type of reactions [50], enantioinduction is not due to repulsive interactions between catalysts and substrates, but rather to the good 3D geometrical fit between the polar functionalities of the catalyst and a TS giving the (*R*)-enantiomer of **9**. Apparently, alternative TSs leading to (*S*)-**9** are not matching well the 3D structure of the catalyst, and cannot thus be stabilized/promoted. The *tert*-butyl moiety serves to control the 3D conformation of the catalyst. Furthermore, catalyst/substrates interactions in this TS are limited to the nitro and N-H groups, the nitroalkene β-substituents do not play an obvious role. Indeed, the reaction with nitroalkenes **4** proved to be applicable with very good results to both aromatic and aliphatic substrates (R in Scheme 10). Besides, these considerations justify the remarkable performances of this catalyst with the different nitroalkenes **1**–**4**, as well as the attack of the hydride to the same pro-chiral face [51] of the nitroolefins, irrespective of the nature of the β-substituents (Scheme 11).

## 3. Materials and Methods

Analytical grade solvents and commercially available reagents were used as received, unless otherwise stated. CH_2_Cl_2_ for the catalytic reactions was filtered on a plug of basic alumina before use. Hantzsch esters **10a**–**c** [52,53] and **10d** [40] were prepared following literature procedures. Chiral thiourea **5g** was prepared as reported in the literature, or purchased from commercial sources [41,42,43]. (*E*)-1-Nitro-2-phenyl-1-propene **1a** [4], β-acylamino nitroolefins and β-*t*-butyloxycarbonylamino nitroolefins **2** [40], ethyl *Z*-3-nitro-2-phenylacrylate **3a** [33], and trifluoromethylated nitroalkenes **4** [54], were prepared following literature procedures.

*(S)-(1-Nitropropan-2-yl)benzene* **6a**: in a screw cap 1.5 mL vial, to a solution of (*E*)-1-nitro-2-phenyl-1-propene **1a** (24.5 mg, 0.15 mmol) in dichloromethane (0.3 mL, 0.5 M), catalyst **5g** (7.6 mg, 0.015 mmol, 0.1 equiv) and Hantzsch ester **10d** (69.5 mg, 0.225 mmol, 1.5 equiv) were sequentially added at 0 °C. The vial was saturated with nitrogen and closed with the cap, then the reaction mixture was stirred for 70 h at 0 °C. The resulting mixture was directly purified by column chromatography eluting with diethyl ether/*n*-hexane 1:15 (*v*/*v*), to afford the title compound in quantitative yield (24.6 mg). The enantiomeric excess of **6a** was determined by CSP HPLC analysis (Chiralcel OJ-H, flow = 0.75 mL/min, eluent: *n*-hexane/*i*-PrOH 90:10, λ = 234 nm, t_maj_ = 20.2 min, t_min_ = 22.2 min). The obtained spectroscopic data were in accord with those previously published [32].

*Asymmetric transfer hydrogenation β-amino nitroalkanes* **2**: in a screw cap round bottom vial, to a stirred solution of **2** (0.15 mmol), toluene (510 μL, 0.3 M), catalyst **5g** (3.9 mg, 0.0075 mmol, 0.05 equiv), and Hantzsch ester **10d** (56 mg, 0.18 mmol, 1.2 equiv) were added. The vial was saturated with nitrogen and closed with the cap. The reaction mixture was stirred for 14 h at 40 °C. The resulting mixture was purified by column chromatography to afford product **7**. The spectroscopic data have been previously published [40].

*(S)-N-(2-Nitro-1-phenylethyl)acetamide* **7a**: the title compound was prepared according to the above procedure and purified by silica gel chromatography (eluent ethyl acetate/petroleum ether 4:1). White solid; Yield 97%. The enantiomeric excess was determined by CSP HPLC analysis using a Daicel Chiralcel OJH column; *n*-hexane/2-propanol 9:1; 0.75 mL/min; λ = 254 nm; *t*_R_ (major) = 35.3 min; *t*_R_ (minor) = 42.8 min; ee >99%.

*tert-Butyl (S)-(2-nitro-1-phenylethyl)carbamate* **7b**: the title compound was prepared according to the above procedure and purified by silica gel chromatography (eluent CH_2_Cl_2_/petroleum ether 6:1). White solid; Yield 90%. The enantiomeric excess was determined by CSP HPLC analysis using a Daicel Chiralcel OJH column; *n*-hexane/2-propanol 9:1; 0.75 mL/min, λ = 254 nm; *t*_R_ (major) = 22.6 min; *t*_R_ (minor) = 25.7 min; ee >99%.

*Asymmetric transfer hydrogenation of β-trifluoromethyl nitroalkenes* **4**: in a screw cap round bottom vial, to a solution of nitroalkene **4** (0.15 mmol) in trifluorotoluene (0.5 mL, 0.3 M), catalyst **5g** (7.6 mg, 0.015 mmol, 0.1 equiv), and Hantzsch ester **10d** (56 mg, 0.18 mmol, 1.2 equiv) were added at −20 °C. The reaction mixture was stirred for 24 h at −20 °C. The resulting mixture was purified by column chromatography eluting with diethyl ether/*n*-hexane 2:50 (*v*/*v*), to afford the trifluoromethyl nitroalkane **9**.

*(R)-1,1,1-Trifluoro-2-phenyl-3-nitropropane* **9a**: in a screw cap round bottom vial, to a solution of nitroalkene **4a** (870 mg, 4.0 mmol) in trifluorotoluene (12 mL, 0.3 M), catalyst **5g** (202 mg, 0.4 mmol, 0.1 equiv) and Hantzsch ester **10d** (1.51 g, 4.8 mmol, 1.2 equiv) were added at −20 °C. The reaction mixture was stirred for 24 h at −20 °C. The resulting mixture was purified by column chromatography eluting with diethyl ether/*n*-hexane 2:50 (*v*/*v*), to afford the trifluoromethyl nitroalkane **9a**. The obtained spectroscopic data were in accordance with those previously published [39]. Yield: 94% (823 mg); pale yellow oil; The enantiomeric excess was determined by CSP HPLC analysis using a Daicel Chiralcel OJH column; *n*-hexane/2-propanol 9:1; 0.75 mL/min; λ = 254 nm. *t*_R_ (major) = 24.4 min; *t*_R_ (minor) = 31.0 min; ee 95%.

*Kinetic studies on the asymmetric transfer hydrogenation of β-trifluoromethyl nitroalkene* **4a**: in a screw cap round bottom vial, to a solution of nitroalkene **4a** (21.8 mg, 0.1 mmol), toluene (800 μL, 0.125 M), catalyst **5** (0.01 mmol, 0.1 equiv), and Hantzsch ester **10d** (46.4 g, 0.15 mmol, 1.5 equiv) were added at 0 °C. The mixture was stirred at the same temperature. The reaction evolution was followed by sampling the mixture with a Pasteur pipette, followed by immediate dilution in CDCl_3_, followed by ^19^F-NMR analysis: product **9a**: −69 ppm (d, *J* = 8.5 Hz, 3F); nitroalkene **4a**: −67 ppm (s, 3F).

## 4. Conclusions

To summarize, we have presented a very general methodology for organocatalytic enantioselective transfer hydrogenation reactions of β,β-disubstituted nitroalkenes, making use of a simple thiourea catalyst, and Hantzsch esters as convenient hydrogen donors. It is the first time that a single catalyst can be used with all nitroalkene substrate classes; previous reports [32,33,34,35,36,37] were all focused on specific catalysts tailored for specific substrates. Some kinetic studies confirmed the generally accepted mode of action of this type of catalysts, allowing us to put forward a reasonable transition state model accounting for the remarkable substrate generality. We envision this synthetic platform might be useful for further synthetic applications and other studies related to thiourea based on catalytic asymmetric transformations [55].

## Figures and Tables

**Table 1:**
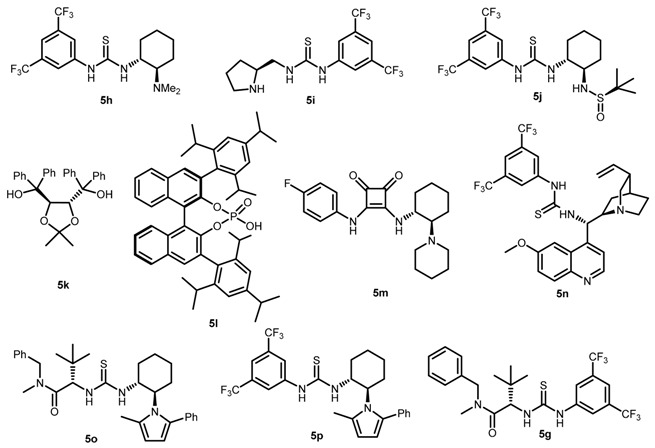
Catalysts screening ^1^.

Entry	Catalyst 5	Conversion ^2^ (%)	ee ^3^ (%)
1	**5h**	97	+12
2	**5i**	80	−9
3	**5j**	>95	+20
4	**5k**	>95	+5
5	**5l**	60	−14
6	**5m**	>95	−17
7	**5n**	87	−24
8	**5o**	>95	+48
9	**5p**	85	−15
10	**5g**	>95	+77

^1^ Conditions: **4a** (0.05 mmol), cat. **5** (10 mol %), toluene (0.5 mL, 0.1 M), **10a** (0.06 mmol, 1.2 equiv), RT, 18–24 h; ^2^ Determined on the crude mixture by ^19^F-NMR analysis; ^3^ Determined by chiral stationary phase HPLC.

**Table 2:**
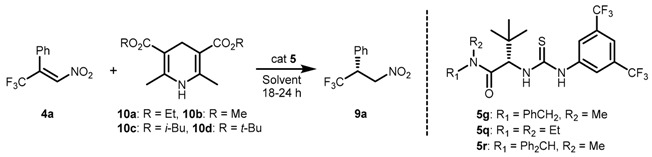
Reaction conditions screening ^1^.

Entry	10	5 (mol %)	Solvent (M)	T (°C)	Conversion ^2^ (%)	ee ^3^ (%)
1	**10a**	**5g** (10)	toluene (0.625)	40	>95	77
2	**10b**	**5g** (10)	toluene (0.625)	40	>95	69
3	**10c**	**5g** (10)	toluene (0.625)	40	>95	70
4	**10d**	**5g** (10)	toluene (0.625)	40	>95	89
5	**10d**	**5g** (10)	CH_2_Cl_2_ (0.13)	40	61	66
6	**10d**	**5g** (10)	MTBE (0.13)	40	60	39
7	**10d**	**5g** (10)	THF (0.13)	40	60	4
8	**10d**	**5g** (10)	toluene (0.625)	−20	>95	94
9	**10d**	**5q** (10)	toluene (0.625)	−20	>95	77
10	**10d**	**5r** (10)	toluene (0.625)	−20	>95	92
11	**10d**	**5g** (10)	PhCF_3_ (0.625)	−20	>95	95
12	**10d**	**5g** (5)	PhCF_3_ (0.625)	−20	>95	92
13	**10d**	**5g** (10)	PhCF_3_ (0.3)	−20	>95	97

^1^ Conditions: **4a** (0.05 mmol), cat. **5**, solvent, **10** (0.06 mmol, 1.2 equiv), 18–24 h; ^2^ Determined on the crude mixture by ^19^F-NMR analysis; ^3^ Determined by chiral stationary phase HPLC.
